# Health professional students’ perceptions regarding their role in tobacco control: findings from the Global Health Professional Students Survey, Pakistan, 2011

**DOI:** 10.1186/1747-597X-9-25

**Published:** 2014-06-23

**Authors:** Syeda Kanwal Aslam, Sidra Zaheer, Kashif Shafique

**Affiliations:** 1 School of Public Health, Dow University of Health Sciences, OJHA Campus, SUPARCO road, Gulzar e Hijri, Karachi, Pakistan; 2 Institute of Health and Wellbeing, Public Health, University of Glasgow, 1-Lilybank Gardens, Glasgow G12 8RZ, UK

## Abstract

**Background:**

An important way of reducing tobacco use is to train the health professional (HP) students to assist in tobacco cessation by educating patients and public. In order to shape their thoughts for the desired role, it is vital to understand their existing perceptions regarding HP’s role in tobacco control. Thus, the aim of our study was to find out the perceptions of Pakistani HP students regarding their future role in tobacco control, and examine factors associated with negative perceptions.

**Methods:**

Secondary data analysis of the Global Health Professional Students Survey, Pakistan, 2011 was performed. Study population included 3445 health professional students in third year of graduate level programs. The dependent variable (perceptions of HP students), was developed using four questions from the survey. Students who did not regard HP’s role in tobacco control were labeled as having negative perceptions. Logistic regression analyses were conducted to analyze association between HP students’ perceptions and various socio-demographic, attitudinal and knowledge related factors; and were reported as adjusted odds ratios with 95% confidence interval.

**Results:**

We found that 44.8% (n = 1542) of students do not regard HPs as role model for their patients and public, and perceive that HPs do not play an important part in patient’s quitting tobacco use. These negative perceptions were associated with male sex (OR = 1.25, 95% CI 1.02 – 1.53, p value 0.028), and poor knowledge about tobacco cessation techniques (OR = 1.32, 95% CI 1.12 – 1.55, p value < 0.001). Negative perceptions were also associated with their attitudes towards ban on: tobacco advertisements (OR = 1.67, 95% CI 1.13 – 2.48, p value 0.010); and tobacco use at public places (OR = 1.60, 95% CI 1.26 – 2.03, p value < 0.001).

**Conclusion:**

The role of HPs for tobacco control is fairly under-perceived by HP students, and the undesired negative perceptions are associated with male sex, poor knowledge about tobacco use cessation techniques and negative attitudes towards legislative control. A comprehensive approach, focusing on these aspects should be adopted to train HPs, in order to utilize them as an effective manpower for tobacco control.

## Background

Tobacco use causes considerable morbidity and mortality worldwide; and tobacco epidemic continues to burden developing countries [1]. Current tobacco use trends threaten to cause over 8 million annual deaths worldwide by the year 2030, and more than two third of these are projected to occur in low and middle income countries [1]. Prevalence of tobacco use in Pakistan is 17% (28% for males, and 5.4% for females) [2]. In terms of Pakistan’s compliance with the Framework Convention on Tobacco control, tobacco use is not banned at all public places, and there are no effective bans on marketing tobacco products. The country did not host any national mass media campaign for last several years [3]. These findings highlight the poor scenario related to tobacco use and its’ legislative control in Pakistan.

An important way of reducing the burden of tobacco use is to train the HPs about tobacco use cessation techniques (TUCT). This will assist them in educating patients and public for tobacco cessation [4-6]. They form the very core of tobacco cessation services that may curb the tobacco related burden in the long run [7]. Several health agencies underscore the importance of training undergraduate HP students to serve as an important manpower for tobacco control in future [8]. Moreover, the need to reduce tobacco use prevalence amongst health professionals, in order to utilize them as the role model for the society; has also been suggested [9].

Despite the increasing prevalence of tobacco use, and suggested measures to equip HPs; the HPs are not trained to assist patients and public in tobacco cessation efforts. To elaborate, firstly, most HPs in our context do not assist their patients in tobacco cessation efforts; they do not receive adequate training in this regard to shape them for their assigned role; they do not perceive HP as a role model for the patients and the public at large, and importantly are themselves the victims of tobacco use [9,10]. To the best of our knowledge, incorporation of formal training on tobacco cessation in education curricula is very scarce, and is almost negligible in countries like Pakistan [9].

These shortcomings are rectifiable, and evidence suggests that formal and informal trainings on tobacco control may be used to shape the attitudes of HP students in order to improve their services in tobacco control [6,11]. Therefore, it is important to understand factors which are associated with negative perceptions amongst HP students. The aim of our study is to find out the perceptions of Pakistani HP students regarding their future role in tobacco control, and identify factors associated with negative perceptions amongst them.

## Methods

Data source: We utilized data of Global Health Professional Students Survey (GHPSS) Pakistan, 2011 for this analysis, which was conducted in 2011 [12]. GHPSS is a standardized school based survey developed by the World Health Organization (WHO), Center of Disease Control and Prevention (CDC), and the Canadian Public Health Association (CPHA), to collect data in all WHO member states.

Sampling method and sample size: GHPSS has a standardized methodology for selecting participants and uniform data processing procedures. Multistage sample design was used, and medical, dental and pharmacy institutes were selected proportional to enrollment size. Classes were chosen randomly within selected institutes, and all students in selected classes were eligible for participation. In accordance with CDC and WHO methodology, sample size was calculated using a precision level of ± 5%. It was estimated that a minimum of 1500 completed student interviews will be needed. The countries use this information to determine sample size of schools and students [13]. Participants consisted of third year students pursuing graduate level degrees in dentistry, medicine, and pharmacy. The survey was conducted during regular class sessions. It used an anonymous, self-administered core questionnaire for data collection. Details of GHPSS are available on CDC website [14].

Ethics and consent: It follows local procedures at each country level, for obtaining ethical review and consent. Specific information about GHPSS Pakistan, in this regard is not available.

The questions selected for this study included information on demographics, tobacco use, attitudes and perceptions regarding tobacco control, and training received regarding patient counseling on tobacco use cessation techniques (TUCT). The dependent variable is perceptions of HP students about their future role in tobacco control, which was developed using four questions from the survey:

1. “Do HPs serve as a role model for their patients and the public?”

2. “Do HPs have a role in giving advice or information about tobacco use cessation to patients?”

3. “Should HPs routinely advise their patients who use tobacco to quit using it?”

4. “Are a patient’s chances of quitting tobacco increased if a HP advises him or her to quit?”

Students who answered “yes” to all four questions were coded as having positive perceptions about the HP’s role and, all others were coded as having negative perceptions. Details of all variables in the study are mentioned in Coding plan (Additional file 1: Table S1).

### Data analysis

The analysis was conducted using SAS version 9.1.3. We used complex survey data analysis for multistage cluster sampling design used in GHPSS. To adjust for cluster sampling, primary sampling units, final weights, and strata were used. We used Chi square tests to determine the statistical significance of the relation between the outcome variable, “perceptions of HP students about their future role in tobacco control” and the independent variables: sex, age, use of any form of tobacco, use of any form of tobacco at institute, knowledge about institute’s official policy on tobacco ban, viewpoint about tobacco ban enforcement at institute, knowledge about TUCT, formal training on TUCT, attitude towards ban on tobacco sale to adolescents < 18 years of age, attitude towards ban on tobacco advertisements, and attitude towards ban on tobacco use at public places. Univariate and Multivariate logistic regression analyses were conducted to report Odds Ratios and 95% Confidence Intervals.

## Results

The study sample included 4,253 undergraduate HP students, from 8 dental (n = 400), 21 medical (n = 2746), and 15 pharmacy (n = 1107) institutions of Pakistan. We constructed the final dataset using information from 3445 participants, after excluding 808 participants due to missing data. The overall students’ response rates were 70.3%, 69.2%, and 70.4% for dental, medical, and pharmacy programs respectively.

We found that 22.0% of HP students (Dental = 15.4, Medical = 23.1, Pharmacy = 21.8) did not perceive HPs as role model for the patients and public. Further, 12.4% of the HP students (Dental = 9.0, Medical = 11.4, Pharmacy = 16.8) perceived that HPs have no role in giving advice about tobacco use cessation techniques to patients. And, 19.6% of them (Dental = 17.6, Medical = 20.2, Pharmacy = 18.8) perceived that there is no improvement in chances of quitting tobacco if a HP advises his/her patients to quit. Furthermore, 14.8% of HP students (Dental = 13.3, Medical = 13.8, Pharmacy = 18.1) opinionated that HPs should not routinely advise their patients who use tobacco to quit using it. See Table 1.

**Table 1:** Descriptive statistics about HP’s perceptions according to their academic background: GHPSS 2011, Pakistan (n = 3445)

**Perception**		**Dental n (%)**	**Medical n (%)**	**Pharmacy n (%)**	**p-value**
**Do HPs serve as “role models” for their patients and the public?**	Yes	274 (84.6)	1748 (76.9)	664 (78.2)	0.008
	No	50 (15.4)	524 (23.1)	185 (21.8)	
**Do HPs have a role in giving advice or information about tobacco use cessation to patients?**	Yes	295 (91.0)	2014 (88.6)	706 (83.2)	< 0.001
	No	29 (9.0)	258 (11.4)	143 (16.8)	
**Are a patient’s chances of quitting tobacco use increased if a HP advises him or her to quit?**	Yes	267 (82.4)	1813 (79.8)	689 (81.2)	0.437
	No	57 (17.6)	459 (20.2)	160 (18.8)	
**Should HPs routinely advise their patients who use tobacco to quit using it?**	Yes	281 (86.7)	1958 (86.2)	695 (81.9)	0.007
	No	43 (13.3)	314 (13.8)	154 (18.1)	

Overall 44.8% (n = 1542) of students had negative perception about their future role in tobacco control. Sex (p value < 0.001); use of any form of tobacco (p value < 0.001); tobacco use at institute (p value < 0.001); knowledge about TUCT (p value < 0.001); and formal training on TUCT (p value 0.004) were statistically significantly associated with perceptions about their future role in tobacco control. Attitudes regarding ban on: sale of tobacco to adolescents under 18 years of age (p value < 0.001); tobacco advertisements (p value < 0.001); and tobacco use at all public places (p value < 0.001); were also statistically significantly associated with negative perceptions. Age and students’ knowledge about official policy of their institute on tobacco ban were not significantly associated with negative perceptions. See Table 2.

**Table 2:** Descriptive characteristics of health professional students regarding perceptions about HP’s role in tobacco control, Pakistan 2011 (n = 3445)*

**Characteristics**	**Total**	**Positive perceptions n (%)**	**Negative perceptions n (%)**	p-value**
**Sex**				
Female	2204	1275 (67.0)	929 (60.2)	< 0.001
Male	1241	628 (33.0)	613 (39.8)	
**Age in years (categorical)**				
19-24	3360	1861 (97.8)	1499 (97.2)	0.397
25-29	70	36 (1.9)	34 (2.2)	
≥ 30	15	6 (0.3)	9 (0.6)	
**Use of any form of tobacco**				
No	2923	1658 (87.1)	1265 (82.0)	< 0.001
Yes	522	245 (12.9)	277 (18.0)	
**Use of any form of tobacco at institute**				
I have never used	2056	1216 (63.9)	840 (54.5)	< 0.001
No	144	82 (4.3)	62 (4.0)	
Yes	1245	605 (31.8)	640 (41.5)	
**Knowledge about official policy on tobacco ban**				
No official policy	1609	883 (46.4)	726 (47.1)	0.690
Yes, for at least institute or clinics	1836	1020 (53.6)	816 (52.9)	
**Viewpoint about ban enforcement at institute**				
Yes	1102	622 (32.7)	480 (31.1)	0.033
No	1364	717 (37.7)	647 (42.0)	
Institute has no official policy	979	564 (29.6)	415 (26.9)	
**Knowledge about tobacco use cessation techniques**				
Good knowledge	832	509 (26.7)	323 (20.9)	< 0.001
Poor knowledge	2613	1394 (73.3)	1219 (79.1)	
**Formal training on tobacco use cessation techniques**				
Yes	835	497 (26.1)	338 (21.9)	0.004
No	2610	1406 (73.9)	1204 (78.1)	
**Attitude towards ban on tobacco use**				
Ban on tobacco sale to adolescents < 18 years				
Yes	3099	1766 (92.8)	1333 (86.4)	< 0.001
No	346	137 (7.2)	209 (13.6)	
Ban on tobacco advertisements				
Yes	3116	1789 (94.0)	1327 (86.1)	< 0.001
No	329	114 (6.0)	215 (13.9)	
Ban on tobacco use at public places				
Yes	2820	1655 (87.0)	1165 (75.6)	< 0.001
No	625	248 (13.0)	377 (24.4)	
**Health professionals**				
Dental	324	209 (11.0)	115 (7.5)	0.002
Medical	2272	1240 (65.2)	1032 (66.9)	
Pharmacy	849	454 (23.8)	395 (25.6)	

*Final dataset was constructed using information from 3445 participants, after excluding 572 participants due to missing information, and 236 participants due to younger age reported (<18 years old).

**The p value has been calculated using Chi square test.

Univariate analyses indicate that HP students were more likely to have negative perceptions about their future role in tobacco control if they: were males, used any form of tobacco (OR = 1.52, 95% CI 1.12 – 2.05); used tobacco at their institute, and had poor knowledge about TUCT. They were also more likely to have negative perceptions if they disagreed with having a ban on: tobacco sale to adolescents under 18 years of age (OR = 1.94, 95% CI 1.46 – 2.58); tobacco advertisements (OR = 2.55, 95% CI 1.93 – 3.36); tobacco use at all public places (OR = 2.14, 95% CI 1.72 – 2.67). Negative perceptions among students were not statistically significantly associated with age, no tobacco use at institute among tobacco users, knowledge about their institute’s policy on tobacco ban, academic background, their viewpoint on ban enforcement at institute, and formal training on TUCT. See Table 3.

**Table 3:** Factors associated with students’ perceptions regarding HP’s role in tobacco control (n = 3445)

**Characteristics**	**Univariate analysis**		**Multivariate analysis**	
	**OR**^**a **^**(95% CI)**	**p-value**	**OR**^**b **^**(95% CI)**	**p-value**
**Sex**				
Female	1		1	
Male	1.38 (1.10 - 1.74)	0.005	1.25 (1.02 - 1.53)	0.028
**Age in years (categorical)**				
19-24	1			
25-29	1.15 (0.44 - 2.95)	0.771	0.99 (0.41 - 2.41)	0.993
≥ 30	1.61 (0.52 - 4.94)	0.400	1.37 (0.47 - 3.99)	0.554
**Use of any form of tobacco**				
No	1		1	
Yes	1.52 (1.12 - 2.05)	0.006	1.09 (0.83 - 1.42)	0.534
**Use of any form of tobacco at institute**				
I have never used	1		1	
No	1.19 (0.65 - 2.18)	0.559	0.73 (0.42 - 1.25)	0.254
Yes	1.47 (1.16 - 1.86)	0.001	1.25 (0.97 - 1.60)	0.073
**Knowledge about official policy on tobacco ban**				
No	1		1	
Yes	0.96 (0.78 - 1.18)	0.723	0.91 (0.72 - 1.14)	0.421
**Viewpoint about ban enforcement at institute**				
Yes	1		1	
No	1.17 (0.93 - 1.48)	0.161	1.08 (0.79 - 1.48)	0.599
Institute has no official policy	0.95 (0.74 - 1.21)	0.691	0.85 (0.64 - 1.13)	0.275
**Knowledge about tobacco use cessation techniques**				
Good	1		1	
Poor	1.4 1 (1.15 - 1.71)	< 0.001	1.32 (1.12 - 1.55)	< 0.001
**Formal Training on tobacco use cessation techniques**				
Yes	1		1	
No	1.24 (0.94 - 1.64)	0.125	1.23 (0.96 - 1.57)	0.092
**Attitude towards ban on tobacco use**				
Ban on tobacco sale to adolescents < 18 years				
Yes	1		1	
No	1.94 (1.46 - 2.58)	< 0.001	1.39 (0.99 - 1.95)	0.052
Ban on tobacco advertisements				
Yes	1		1	
No	2.55 (1.93 - 3.36)	< 0.001	1.67 (1.13 - 2.48)	0.010
Ban on tobacco use at all public places				
Yes	1		1	
No	2.14 (1.72 - 2.67)	< 0.001	1.60 (1.26 - 2.03)	< 0.001
**Health Professionals**				
Dental	1		1	
Medical	1.29 (0.67 - 2.50)	0.436	1.33 (0.72 - 2.47)	0.353
Pharmacy	1.37 (0.67 - 2.76)	0.379	1.42 (0.75 - 2.71)	0.278

OR^a^ = unadjusted odds ratios.

OR^b^ = odds ratios adjusted for all independent variables, CI = confidence intervals.

We found that students were more likely to have negative perceptions if they: were males or had poor knowledge about TUCT,; after adjusting for all covariates in multivariate analysis, including sex, age, use of any form of tobacco, tobacco use at institute, knowledge about institute’s official policy on tobacco ban, viewpoint about ban enforcement at institute, knowledge about TUCT, formal training on TUCT, attitude towards ban on tobacco sale to adolescents under 18 years of age, attitude towards ban on tobacco advertisements, attitude towards ban on tobacco use at public places and their academic background. Furthermore, they were also more likely to have negative perceptions if they disagreed with having a ban on: tobacco advertisements (OR = 1.67, 95% CI 1.13 – 2.48) and tobacco use at all public places (OR = 1.60, 95% CI 1.26 – 2.03). See Table 3.

## Discussion

This study found that almost half of the students had negative perceptions regarding HP’s role in tobacco control, which were associated with male sex, having poor knowledge about TUCT, and not receiving any formal training on TUCT. Negative perceptions were also associated with their attitudes towards ban on: tobacco advertisements and tobacco use at public places.

Our finding regarding the negative perceptions among HP students indicates that they do not regard HPs as role model for their patients and public, and perceive that HPs do not play an important part in patient’s quitting tobacco use. In our study, we report the combined proportion of negative perceptions among HP students. The proportion of negative perceptions for each item question is comparable with negative perceptions among HP students of other developing countries like India, Iran, and Mexico; and higher as compared to countries like Saudi Arabia, Bangladesh, and Nepal [15]. The finding of having almost half of the students negatively perceiving HP’s role, predicts the future role these students from developing world might play in tobacco control. It highlights an important behavioral aspect: If they do not regard HPs as an important taskforce in tobacco control, they might themselves miss the opportunities to advise their patients and the public at large. Abdullah AS et al. report that positive beliefs of physicians regarding their role are associated with counseling attempts [16]. The need for the professional trainings to address beliefs, attitudes, and confidence levels in relation to smoking cessation has also been highlighted by other studies [10].

We attempted to examine the factors that may be associated with these undesired perceptions. Firstly, male students were more likely to have negative perceptions. Although the effect size of sex in our study was modest, but the finding is comparable with other developing countries like Bangladesh and Iran [15]. Previous studies have also highlighted that male HP students are more likely to be smokers, and thus have poorer aspirations towards their service as role model for patients and society at large [9]. However, our findings suggest an association with male sex, regardless of tobacco use. This highlights the need to focus on males while attempting to change perceptions among HP students; and may direct future research focus on sex based differences in perceptions regarding role modeling.

Negative perceptions among HP students were also associated with poor knowledge base regarding TUCT. Students, who either had poor prior knowledge or received no formal training in tobacco cessation, were more likely to have negative perceptions. The effect size of prior knowledge on tobacco cessation was modest, but several studies report its association with a perceived role in cessation counseling [17]. The need to effectively train the future workforce cannot be denied, and has been emphasized by the health experts for more than a decade now [18-20]. Unfortunately, we have not been able to implement effective measures to equip our future workers with the required skills. In developing countries, incorporation of formal training on tobacco cessation in education curricula is very scarce, and is almost null in countries like Pakistan [9].

HP students’ attitude towards legislative control of tobacco use is an important consideration. It is of concern that students who did not agree with having a ban on tobacco advertisements and smoking at public places, were most likely to have negative perceptions. These negative attitudes towards tobacco ban were the strongest predictors of negative perceptions in our study. Several other studies have also reported skepticism of HP students towards tobacco ban, associated with their knowledge on harmful effects of smoking, and their own smoking status [17,21]. Thus, one way of gaining the HP students’ support for tobacco control is to improve their knowledge about harmful effects of tobacco, further underscoring the need of HP training on TUCT.

This study reports association of various factors associated with wrong perceptions of HP students regarding their role in tobacco control in Pakistani context. Further research will be needed to confirm these findings, and determine whether these findings can be used to advocate the need to mold HP students’ perceptions. Our study has various limitations; firstly, GHPSS focuses on HP students in third year of their professional education. Clinical rotations usually begin during the third year of their professional education. It is possible, that their perceptions and attitudes might change over time, as they gain more knowledge through the completion of their professional courses; and gain more insight into the context as they interact further with the patients during later years of education. Secondly, it uses yes or no option for assessing perceptions and attitudes instead of Likert scale or qualitative methods. Thirdly, we did not deal with the non-respondent rate. Lastly, the data are self-reported but consistent evidence indicates that results obtained from such data are reliable [22].

## Conclusion

The role of HPs for tobacco control is fairly under-perceived by HP students and the negative perceptions are mainly associated with male sex, poor knowledge about TUCT and negative attitudes towards legislative control. A comprehensive approach, focusing on these aspects should be adopted to train HPs, in order to utilize them as an effective manpower for tobacco control in developing world.

## Competing interest

The authors declare that they have no competing interests.

## Authors’ contribution

KS conceived the idea, and supervised the study. SK and SZ designed the study; SZ carried out statistical analyses; all authors contributed to interpreting the results; SK drafted the manuscript; and all authors approved the final manuscript.

## Supplementary Material

Additional file 1: Table S1 Coding plan for the selected study variables. Click here for file
