# Supplementary material for: Health professional students’ perceptions regarding their role in tobacco control: findings from the Global Health Professional Students Survey, Pakistan, 2011
Source: Subst Abuse Treat Prev Policy. 2014 Jun 23;9:25. doi: 10.1186/1747-597X-9-25 (PMC4078011; doi:10.1186/1747-597X-9-25)
Supplement: Additional file 1: Table S1 — Coding plan for the selected study variables. [file 1747-597X-9-25-S1.docx]

| **Additional file 1: Table S1: Coding plan for the selected study variables** | | | | |
| --- | --- | --- | --- | --- |
| **Variable** | **Question In Codebook** | | **Response Options** | **Labels Used After Recoding** |
| **Outcome Variable** |  | |  |  |
| **Perception of HP students regarding HP's role in tobacco control** | 1. Do health professionals serve as "role models" for their patients and the public? | | 1= Yes | Students who answered Yes to all four questions were recoded as Positive perception (= 1) and all others were recoded as Negative perception (= 0). |
|  |  |  | 2= No |  |
|  |  |  |  |  |
|  |  | |  |  |
|  | 2. Do health professionals have a role in giving advice or information about tobacco use cessation to patients? | | 1= Yes |  |
|  |  |  | 2= No |  |
|  |  |  |  |  |
|  |  | |  |  |
|  | 3. Should health professionals routinely advise their patients who use tobacco to quit using it? | | 1= Yes |  |
|  |  |  | 2= No |  |
|  |  |  |  |  |
|  |  | |  |  |
|  | 4. Are a patient's chances of quitting tobacco use increased if a health professional advises him or her to quit? | | 1= Yes |  |
|  |  |  | 2= No |  |
|  |  |  |  |  |
|  |  |  |  |  |
| **Independent Variables** | |  |  |  |
| **Sex** | What is your gender? | | 1= Female | 0= Female |
|  |  | | 2= Male | 1= Male |
|  |  | |  |  |
| **Age in years (categorical)** | How old are you? | | 1= 14 years old or younger | 0= 19 - 24 |
|  |  | | 2= 15 to 18 years | 1= 25 - 29 |
|  |  | | 3= 19 to 24 years | 2= ≥ 30 |
|  |  | | 4= 25 to 29 years |  |
|  |  | | 5= 30 years or older | (Those who reported to be <18 years old were excluded). |
|  |  | |  |  |
| **Use of any form of tobacco** | 1. During the past 30 days (one month), on how many days did you smoke cigarettes? | | 1= 0 days | Students who answered 0 days to all three questions were recoded as No (= 0) and all others were recoded as Yes (= 1). |
|  |  |  | 2= 1 or 2 days |  |
|  |  |  | 3= 3 to 5 days |  |
|  |  |  | 4= 6 to 9 days |  |
|  |  |  | 5= 10 to 19 days |  |
|  |  |  | 6= 20 to 29 days |  |
|  |  |  | 7= All 30 days |  |
|  |  | |  |  |
|  | 2. During the past 30 days (one month), on how many days did you use shish (Hukka)? | | 1= 0 days |  |
|  |  |  | 2= 1 or 2 days |  |
|  |  |  | 3= 3 to 5 days |  |
|  |  |  | 4= 6 to 9 days |  |
|  |  |  | 5= 10 to 19 days |  |
|  |  |  | 6= 20 to 29 days |  |
|  |  |  | 7= All 30 days |  |
|  |  | |  |  |
|  | 3. During the past 30 days (one month), on how many days did you use chewing tobacco, snuff, bidis, cigars, or pipes (excluding cigarettes & shisha (Hukka)? | | 1= 0 days |  |
|  |  |  | 2= 1 or 2 days |  |
|  |  |  | 3= 3 to 5 days |  |
|  |  |  | 4= 6 to 9 days |  |
|  |  |  | 5= 10 to 19 days |  |
|  |  |  | 6= 20 to 29 days |  |
|  |  |  | 7= All 30 days |  |
|  |  | |  |  |
| **Use of any form of tobacco at institute** | 1. Have you ever smoked cigarettes inside school buildings during the past year? | | 1= I have never smoked cigarettes | Those who answered I have never used to both questions were recoded as I have never used (= 0). Those who answered No to both questions and; I have never used to at least 1 question and No to other question were recoded as No (= 1), Those who answered Yes to at least one questions were recoded as Yes (= 2). |
|  |  |  | 2= Yes |  |
|  |  |  | 3= No |  |
|  |  | |  |  |
|  | 2. Have you ever used chewing tobacco, snuff, bidis, cigars, or pipes inside school buildings during the past year? (excluding cigarettes & shisha or Hukka) | | 1= I have never used all |  |
|  |  |  | 2= Yes |  |
|  |  |  | 3= No |  |
|  |  | |  |  |
| **Knowledge about official policy on tobacco ban** | Does your school have an official policy banning smoking (cigarettes, shisha (Hukka), or other tobacco products) in school buildings and clinics? | | 1= Yes, for school buildings only | No official policy response remained same (= 0). All other options were recoded as Yes, for at least institute or clinics (= 1). |
|  |  |  | 2= Yes, for clinics only |  |
|  |  |  | 3= Yes, for both |  |
|  |  |  | 4= No official policy |  |
|  |  | |  |  |
| **Viewpoint about ban enforcement at institute** | Is your school's official policy putting a ban on smoking cigarettes, shisha (Hukka), or other tobacco products within school buildings enforced? | | 1= Yes, policy is enforced | 0= Yes |
|  |  |  | 2= No, policy is not enforced | 1= No |
|  |  |  | 3= School has no official policy | 2= Institute has no official policy |
|  |  | |  |  |
| **Knowledge about tobacco use cessation techniques** | 1. During your medical, dental, nursing or pharmacy school training, did you learn that it is important to provide educational/ cessation materials to stop tobacco use to patients who want to quit? | | 1= Yes | Those who answered Yes to all three questions were recoded as Good knowledge (= 0); and all others were recoded as Poor knowledge (= 1). |
|  |  |  | 2= No |  |
|  |  |  |  |  |
|  |  |  |  |  |
|  |  | |  |  |
|  | 2. Have you ever heard of using nicotine replacement therapies in tobacco cessation programs (such as nicotine patch, or gum or Champex)? | | 1= Yes |  |
|  |  |  | 2= No |  |
|  |  |  |  |  |
|  |  |  |  |  |
|  |  | |  |  |
|  | 3. Have you ever heard of using antidepressants in tobacco cessation programs (such as bupropion, or Zyban)? | | 1= Yes |  |
|  |  |  | 2= No |  |
|  |  |  |  |  |
|  |  |  |  |  |
|  |  | |  |  |
| **Formal training on tobacco use cessation techniques** | During your medical, dental, nursing or pharmacy training, have you ever received any formal training in tobacco use cessation approaches to use with patients? | | 1= Yes | 0= Yes |
|  |  |  | 2= No | 1= No |
|  |  |  |  |  |
|  |  | |  |  |
| **Attitude towards ban on tobacco use** | | |  |  |
| **Ban on tobacco sale to adolescents < 18 years** | Should tobacco (cigarettes, shisha (Hukka), or other tobacco products) sales to adolescents 9 persons younger than 18 years old) be banned? | | 1= Yes | 0= Yes |
|  |  |  | 2= No | 1= No |
|  |  |  |  |  |
|  |  |  |  |  |
|  |  | |  |  |
| **Ban on tobacco advertisements** | Should there be a complete ban of the advertising of tobacco products (cigarettes, shisha (Hukka), or other tobacco products)? | | 1= Yes | 0= Yes |
|  |  |  | 2= No | 1= No |
|  |  |  |  |  |
|  |  | |  |  |
| **Ban on tobacco use at public places** | 1. Should smoking any tobacco product (cigarettes, shisha (Hukka), or other tobacco products) be banned in restaurants? | | 1= Yes | Students who answered Yes to all four questions were recoded as Yes (= 0) and all others were recoded as No (= 1). |
|  |  |  | 2= No |  |
|  |  | |  |  |
|  | 2. Should smoking any tobacco product (cigarettes, shisha (Hukka), or other tobacco products) be banned in entertainment places/internet cafes? | | 1= Yes |  |
|  |  |  | 2= No |  |
|  |  | |  |  |
|  | 3. Should smoking any tobacco product (cigarettes, shisha (Hukka), or other tobacco products) be banned in cafes? | | 1= Yes |  |
|  |  |  | 2= No |  |
|  |  | |  |  |
|  | 4. Should smoking any tobacco product (cigarettes, shisha (Hukka), or other tobacco products) be banned in all enclosed public places? | | 1= Yes |  |
|  |  |  | 2= No |  |
